# Alterations in Concentration/Activity of Superoxide Dismutases in Context of Obesity and Selected Single Nucleotide Polymorphisms in Genes: *SOD1*, *SOD2*, *SOD3*

**DOI:** 10.3390/ijms21145069

**Published:** 2020-07-17

**Authors:** Łukasz Lewandowski, Marta Kepinska, Halina Milnerowicz

**Affiliations:** Department of Biomedical and Environmental Analyses, Faculty of Pharmacy, Wroclaw Medical University, Borowska Street 211, 50-556 Wrocław, Poland; marta.kepinska@umed.wroc.pl (M.K.); halina.milnerowicz@umed.wroc.pl (H.M.)

**Keywords:** metals, obesity, oxidative stress, single nucleotide polymorphisms, superoxide dismutase isozymes

## Abstract

Little is known about the contribution of each of the three superoxide dismutase isozymes (SODs) to the total SOD activity in extracellular fluids. This study was aimed to investigate the alterations in concentration/activity of (SODs) in plasma, in context of sex, obesity, exposition to cigarette smoke, and genotypic variability of five selected single nucleotide polymorphisms (SNPs) in genes *SOD1*, *SOD2*, *SOD3*. Men showed higher SOD1 concentration, lower SOD3 concentration and higher total antioxidative capacity (TAC) values. Intersexual variability was observed in concentration of copper, zinc, and cadmium. The obese showed higher total oxidative capacity regardless of sex. An increase in SOD2 activity was coexistent with obesity in men, and exposition to cigarette smoke in non-obese individuals. Additionally, in state of this exposition, Cu,Zn-SOD contribution to the total SOD was lower. Interestingly, over 90% of the obese were of C/T genotype of rs4880 (*SOD2*). Non-obese of T/T genotype (rs4880) were of lower total SOD activity due to decrease in both Cu,Zn-SOD and Mn-SOD activities. SNP rs2234694 was associated with differences in concentration of SODs, depending on obesity status. Correlations indicate that both TAC and SODs, together, may adapt to insulin resistance and inflammation-derived oxidative stress found in obesity. This topic should be further investigated.

## 1. Introduction

The demand to thoroughly understand the risk factors for developing obesity stems from the worldwide increase in prevalence of this disease [1], especially in the recent years. It is commonly claimed that oxidative stress, coexisting with dyslipidemia, aberrant immunological response, disorders of the electrolyte balance, mitochondrial dysfunction, increased secretion of proinflammatory adipokines [2,3,4,5], and activation of inflammasomes and toll-like receptors contribute to the state of meta-inflammation, thus disorders such as insulin resistance, type 2 diabetes, and atherosclerosis often co-existent with obesity [6].

Contemporary medicine links oxidative stress with insulin resistance [7,8,9]. In a state of normoglycemia, glucose is metabolized mainly in two pathways: glycolysis and pentose phosphate pathway and reactive oxygen species (ROS) are sufficiently neutralized by the antioxidative system, comprised of low-molecular weight compounds (e.g., glutathione, bilirubin, uric acid, ascorbic acid, D-α-tocopherols) and high-molecular-weight compounds (enzymatic antioxidants). However, in the state of hyperglycemia, mitochondrial dysfunction leads to overproduction of electron donors (mainly NADH+H+). Excessive ROS generation and deficiency in antioxidative capacity caused by inactivation of enzymatic antioxidants, partly in the process of enzyme glycation, lead to disorders in lipid and carbohydrate metabolism and oxidative damage in proteins and DNA [10,11]. Moreover, superoxide anion, through oxidative modification, decreases the activity of glyceraldehyde 3-phosphate dehydrogenase (GAPDH) [12], which leads to intensification of carbohydrate metabolism via alternative pathways: polyol (sorbitol) and hexosamine [13,14]. Activation of protein kinase C by diacylglycerol and advanced glycation end products formed from glyceraldehyde 3-phosphate (in the state of GAPDH activity deficiency) causes phosphorylation (inactivation) of insulin receptor substrate-1 (IRS-1), therefore leading to insulin resistance [15,16,17]. Moreover, the state of insulin resistance in obesity is promoted by disorders in modulation of the processes of secretion and degradation of insulin (due to hypozincemia) [18,19], glycation of insulin [20], “leptin resistance” causing accumulation of triglycerides in muscle tissue [21,22,23] and disorders in secretion of neuropeptides [24].

This study focuses on superoxide dismutases (SODs), a vital family of enzymatic antioxidants, which neutralize superoxide anion. In humans SOD is found in form of three isozymes: cytosolic SOD1 (gene *SOD1*, chromosome 21q22.11), mitochondrial SOD2 (gene *SOD2*, chromosome 6q25.3), and extracellular SOD3 (gene *SOD3*, chromosome 4p15.2) [25,26]. SOD1 and SOD3 are copper-zinc SODs, whereas SOD2 is a manganese SOD. Interestingly, despite the fact that two of three mentioned SODs are ascribed to the intracellular compartment, it is known that all of them could be found in the extracellular compartment (serum, plasma), which is assumed to be due to migration of intracellular isozymes SOD1 and SOD2 to the extracellular matrix [27,28,29]. The association of SOD with obesity is due to its protective role as an antioxidant [30]. The extracellular SOD, SOD3, due to its abundance on the surface of endothelium, neutralizes the superoxide anion in the extracellular matrix, disrupting the generation of peroxynitrite, one of the mediators of pancreatic β-cell damage [31]. SOD2, found mainly in the mitochondria, by neutralizing superoxide anion, prevents from increased activation of uncoupling proteins (UCPs) by superoxide anion (which is observed in state of chronic hyperglycemia, as found in obesity). As UCPs affect the process of insulin secretion in response to actual glycemia, maintaining proper SOD2 activity is important in context of obesity [32,33,34]. Moreover, SODs protect from the development of diabetes since it was shown that increased expression of SOD1 in pancreatic islet β cells is associated with increased resistance to alloxan-induced diabetes [35] and increased SOD activity leads to increased resistance to streptozocin-induced diabetes [36,37,38,39].

The variability in SOD concentration/activity between individuals may be associated with single nucleotide polymorphisms (SNPs) in genes coding for SOD isozymes. Flekac et al. [40] showed that diabetic individuals of C/C genotype (SNP rs2234694, *SOD1* gene) were characterized of lower total SOD activity. Another SNP, rs1799895 (*SOD3*), is associated with the loss of *SOD3* affinity to heparan sulfate (due to Arg213Gly substitution in the extracellular matrix binding region of SOD3), causing an increased concentration of SOD3 in extracellular fluid [41,42,43]. Individuals of no allele associated with Ile58Thr substitution in SOD3 were characterized of lower SOD activity [44]. One of the most-studied SNPs of the *SOD2* gene, rs4880, associated with Val16Ala substitution and thus conformational changes in the mitochondrial targeting sequence of SOD2 is not only associated with variability of SOD activity in mitochondrial matrix [45], but also variability in total SOD activity and malondialdehyde, leptin and total cholesterol concentration in blood, in both obese and/or non-obese [40,46]. Moreover, it has been shown that genotypic variability of rs4880 may be associated with difference in chances of developing obesity, as the individuals of Val/Val genotype had two-fold increased chance of developing obesity, compared to individuals of Ala/Ala or Ala/Val genotype [47].

This work is the result of reflections on the subject of the influence of a hereditary factor—DNA polymorphism in genes *SOD1*, *SOD2*, *SOD3* on the variability of concentration/activity of SOD isozymes in plasma. The additional factors taken into consideration in this study are sex, obesity, and exposition to cigarette smoke. To our knowledge, apart from two SNPs, rs2234694 (*SOD1*) and rs4880 (*SOD2*), the SNPs investigated in this study (rs5746105, rs927450, rs8192287) have not previously been studied in the context of obesity. Interestingly, regarding extracellular fluid (serum, plasma), the literature covers mostly the topic of changes in total SOD activity. Only a few studies provide information on the activity of individual fractions Cu,Zn-SOD (SOD1 and SOD3) and Mn-SOD (SOD2), which results from the specific nomenclature, suggesting that SOD1 and SOD2 are mainly intracellular enzymes. Determining only the total SOD activity prevents from deeper understanding of how individual SOD isozymes interact to maintain proper antioxidative capacity in the extracellular fluid and if obesity affects this interaction. Moreover, the literature does not thoroughly cover the topic of the association between the concentration of copper and zinc with the alterations in concentration/activity of copper–zinc isozymes SOD1 and SOD3. The aim of this work is to attempt to cover all of these topics in one study. The changes in SOD isozyme concentration/activity are also analyzed in reference to total antioxidative capacity—mainly associated with low-molecular-weight antioxidants, and concentration of malondialdehyde (MDA)—a marker of lipid peroxidation process.

## 2. Results

### 2.1. Intersexual Variability in Concentration/Activity of SOD Isozymes, Total Antioxidative Capacity (TAC) Values, Concentration of Malondialdehyde (MDA) and Metals: Copper, Zinc, and Cadmium

The analysis was performed on values observed in individuals not exposed to cigarette smoke. Descriptive statistics is shown in Table 1. Although no significant differences between women and men were found in values of SOD activity, men were characterized of significantly higher SOD1 concentration (*p* < 0.00001) and lower SOD3 concentration (*p* ≈ 0.0214 and *p* ≈ 0.0643 for SOD3 (ng/mL) and SOD3 (ng/mg total protein), respectively) in plasma. Interestingly, higher total antioxidative capacity (TAC) values (*p* < 0.000001) and MDA concentration (*p* ≈ 0.0085) were observed in men, compared to women.

Significant intersexual variability was also found in values of concentration of copper, zinc, in serum, and cadmium, in full blood. Men were characterized of lower cadmium concentration (*p* ≈ 0.0125) and lower zinc-to-copper ratio (*p* < 0.00001) due to significantly lower copper concentration (*p* ≈ 0.0144) and higher zinc concentration (*p* < 0.00001).

### 2.2. Alterations in Concentration/Activity of Superoxide Dismutase (SOD) Isozymes, TAC Values, Concentration of MDA and Metals: Copper, Zinc, and Cadmium, in Context of Obesity and Exposition to Cigarette Smoke

The analysis in context of obesity was performed on data of individuals not exposed to cigarette smoke. Different observations were made depending on sex. In women (Table 2), no difference was found in concentration/activity of SODs, although the mean specific Cu,Zn-SOD activity (U/mg SOD1+SOD3) was markedly (approximately, 34%) lower (*p* ≈ 0.0814) in obese individuals. Moreover, higher values of TAC (*p* ≈ 0.0222) and MDA (*p* < 0.0001) concentration were observed in obese women, compared to the non-obese. Lower zinc-to-copper ratio and higher concentration of copper (in serum) and cadmium (in full blood) were found in obese women, compared to the non-obese.

In men (Table 3), similarly to women, no difference was found in concentration/activity of SODs, but in the obese, the mean concentration of SOD1 (ng/mL) was approximately 22% higher (*p* ≈ 0.0575), the mean concentration of SOD2 (ng/mL) was approximately 34% lower (*p* ≈ 0.0697), and the mean Cu,Zn-SOD specific activity (U/mg SOD1+SOD3) was approximately 30% lower (*p* ≈ 0.0648), compared to the non-obese. Interestingly, both the activity and specific activity of Mn-SOD were higher in obese men; the *p* values were approximately 0.0015, 0.0006, 0.0145, for variables Mn-SOD (U/L), Mn-SOD (U/g total protein), Mn-SOD (U/mg SOD2), respectively. The TAC values in the obese were higher, although insignificantly (*p* ≈ 0.0581). No significant difference was found in concentration values of MDA, copper, zinc, and cadmium.

The analysis in context of exposition to cigarette smoke was performed regardless of sex due to relatively low count of exposed individuals. In both the non-obese (Table 4) and obese (Table 5), higher cadmium concentration (*p* < 0.0001) was found in individuals exposed to cigarette smoke, compared to the non-exposed. Interesting observations regarding the activity of SODs were made in the control group. In that group, the exposed were characterized of higher Mn-SOD activity (*p* ≈ 0.0146; *p* ≈ 0.0419 in case of Mn-SOD (U/L), Mn-SOD (U/g total protein), respectively) and markedly (approximately 49%), but insignificantly (*p* ≈ 0.0592) lower contribution of Cu,Zn-SOD to the total SOD activity pool. The values of the rest of analyzed parameters seemed unaffected by the exposition status.

### 2.3. Selected Correlations between the Concentration/Activity of SODs and Concentration of Copper, Zinc, and Cadmium, and Other Parameters

Firstly, monotonic (Spearman) correlations between the concentration of copper, zinc, and cadmium, concentration/activity of SODs, TAC values, and MDA concentration, and age, parameters describing lipid and carbohydrate metabolism, insulin resistance, and inflammation were analyzed, as shown in Table 6. Concentration of copper was positively correlated with age, TChol, TG, LDL-Chol, and CRP concentration. Concentration of zinc was positively correlated with TG, LDL-Chol, and glucose concentration, and negatively correlated with HDL-Chol concentration. Cadmium concentration was positively correlated with age and body mass index (BMI). Concentration of SOD1 was positively correlated with TG, insulin concentration and HOMA-IR index, and negatively correlated with HDL-Chol concentration. Total SOD activity was negatively correlated with parameters elevated in obesity: TChol, TG, CRP, insulin concentration, BMI, and HOMA-IR indices. Cu,Zn-SOD activity was negatively correlated with age, TChol, LDL-Chol concentration, and BMI index. Mn-SOD activity was not significantly correlated with any of the mentioned parameters. The contribution of Cu,Zn-SOD to the total SOD activity pool was negatively correlated with age and LDL-Chol concentration, and positively with HDL-Chol concentration. Interestingly, the values of TAC and MDA concentration were both positively correlated with age, TG, CRP, glucose, insulin concentration, BMI index, and negatively correlated with HDL-Chol concentration. Additionally, TAC values, unlike MDA concentration, were positively correlated with HOMA-IR index.

Secondly, correlations between the concentration/activity of SODs, TAC values and concentration of MDA, copper, zinc, and cadmium were analyzed, as shown in Table 7. SOD1 concentration was negatively correlated with SOD2 concentration and positively correlated with SOD3 concentration, total SOD activity, Cu,Zn-SOD activity, contribution of Cu,Zn-SOD to the total SOD activity pool, and TAC values. SOD3 concentration, apart from the mentioned correlation with SOD1 concentration, was positively correlated with Cu,Zn-SOD activity and contribution of Cu,Zn-SOD to the total SOD activity pool. Interestingly, both the Cu,Zn-SOD activity and contribution of Cu,Zn-SOD to the total SOD activity pool were negatively correlated with MDA concentration. Moreover, TAC values were positively correlated with MDA concentration. Both TAC values and MDA concentration were positively correlated with zinc concentration. Surprisingly, no significant correlations were found between the concentration of zinc and concentration/activity of SODs, copper concentration was negatively correlated with total SOD activity and Cu,Zn-SOD activity, and cadmium concentration was positively correlated with Mn-SOD activity.

### 2.4. Genotyping Results—Genotypic Frequency in Context of Obesity

Contingency tables (Table 8) showed no significant differences in genotype frequency, between the obese and non-obese, in SNPs: rs2234694 (*p* ≈ 0.5807), rs5746105 (*p* ≈ 0.2985), rs927450 (*p* ≈ 0.8364), rs8192287 (*p* = 1.0000). Regarding rs2234694, over 80% of individuals were of A/A genotype regardless of obesity status. The T allele of rs5746105 was detected in over 80% of individuals regardless of the obesity status and the C/C genotype was slightly more frequent in obese (15.91%), compared to the non-obese (6.00%). The T allele of rs927450 was found in over 70% of individuals regardless of obesity status and 24.00% and 20.45% of individuals were of C/C genotype in the control and the obese group, respectively. The SNP rs8192287 was ruled out of analysis, as in both control and obese groups all of individuals were of G/G genotype.

Significant (*p* ≈ 0.0015) difference in genotype frequency was found regarding SNP rs4880 (*SOD2*). Dominance of the C/T genotype was observed in the obese group, compared to the control group (93.18% vs. 62.00%). The remaining frequency was more less equally distributed between genotypes: C/C and T/T. The difference in genotype frequency remained significant (*p* ≈ 0.0009) after merging the less frequent genotypes (C/C and T/T) into one group.

### 2.5. Values of SOD Concentration/Activity, TAC, and Concentration of MDA, in Context of Genotypic Variability of the Chosen Single Nucleotide Polymorphisms in Genes: SOD1, SOD2, SOD3

Due to the lack of genotypes other than G/G, SNP rs8192287 (*SOD3*) was not included in this analysis. Two SNPs in this study, rs2234694 (*SOD1*) and rs4880 (*SOD2*), were found to be significantly associated with alterations in concentration/activity of SODs. The observations varied, depending on the obesity status. Descriptive statistics regarding the SNPs, which were not associated with any change in values of the anti(pro-)oxidative parameters assayed for in this study, is shown in Appendix A: Table A1 and Table A2 (rs5746105), Table A3 and Table A4 (rs927450).

Non-obese individuals (control group) of A/C genotype (rs2234694) showed approximately 45% lower SOD3 concentration in plasma (*p* ≈ 0.0165; *p* ≈ 0.0304 in case of SOD3 (ng/mL), SOD3 (ng/mg total protein), respectively) and higher MDA concentration (*p* ≈ 0.0423), compared to individuals of A/A genotype. Lower values of SOD1 concentration (*p* ≈ 0.0215; *p* ≈ 0.0493 in case of SOD1 (ng/mL), SOD1 (ng/mg total protein), respectively) and higher values of SOD2 concentration (*p* ≈ 0.0460 in case of both SOD2 (ng/mL) and SOD2 (ng/mg total protein)) were found in obese individuals of A/C genotype (rs2234694), compared to individuals of A/A genotype. The lack of difference in age, cadmium, and cotinine concentration and BMI between individuals of the two genotypes, in both the control and obese group indicate that age, exposition to cigarette smoke and uneven distribution of body fat (BMI index) are unlikely to be associated with the mentioned findings. Descriptive statistics regarding this SNP (rs2234694) are shown in Table 9 (non-obese) and Table 10 (obese).

As over 90% of obese individuals were of C/T genotype (rs4880), only the control group was included in the analysis in context of this genotype. Non-obese individuals of T/T genotype showed lower SOD3 concentration in plasma (*p* ≈ 0.0663; *p* ≈ 0.0486 in case of SOD3 [ng/mL], SOD3 [ng/mg total protein], respectively), compared to individuals of C/C or C/T genotype. In the same (control) group, total SOD activity, Cu,Zn-SOD activity, and Mn-SOD activity were markedly lower in individuals of C/T genotype. The type I error for comparisons in variables SOD [U/L], SOD [U/g total protein], Cu,Zn-SOD [U/L], Cu,Zn-SOD [U/g total protein], Mn-SOD [U/L], Mn-SOD [U/g total protein] were 0.0007, 0.0005, 0.0202, 0.0189, 0.0539, and 0.0165, respectively. No significant difference in age, cadmium, and cotinine concentration were found, indicating that age and exposition to smoke were unlikely to affect these findings. The difference in BMI values, being on the brink of statistical significance (*p* ≈ 0.0673) suggests a possible association of rs4880 (*SOD2*) with obesity. Descriptive statistics regarding this SNP (rs4880) are shown in Table 11.

### 2.6. Other Findings Regarding Genotypic Variability of Single Nucleotide Polymorphisms (SNPs): rs2234694 (SOD1), rs4880 (SOD2)

An unexpected observation was made regarding the genotypic variability of SNPs rs2234694 (*SOD1*) and rs4880 (*SOD2*), which, in this study, were associated with alterations in concentration/activity of SODs. Interestingly, the significant differences were found in parameters associated with carbohydrate metabolism and insulin resistance (Table 12). The obese of A/C genotype (rs2234694) were characterized of over 70% lower median insulin concentration (*p* ≈ 0.0214) and over 80% lower median HOMA-IR index value (*p* ≈ 0.0218), compared to individuals of A/A genotype. The non-obese of T/T genotype (rs4880) showed higher values of glucose concentration (*p* ≈ 0.0326), insulin concentration (*p* ≈ 0.0369) and HOMA-IR index (*p* ≈ 0.0306), compared to individuals of C/C or C/T genotype, indicating a possible association of rs4880 (*SOD2*) with the state of obesity and insulin resistance. More thorough information on values of basic clinical parameters (lipidogram, glucose, insulin, CRP concentration, HOMA-IR index) can be found in Appendix A (Table A5).

## 3. Discussion

The first issue to be addressed in this discussion is the proper a priori classification of individuals into study groups and the identity of the results of basic parameters used in laboratory diagnostics. The obese group was characterized of higher TG, CRP, glucose, insulin, BMI and HOMA-IR indices, and lower HDL-Chol concentration, compared to the non-obese group, which accounts for the lack of discrepancy between the observed characteristics of both groups and the “typical” clinical picture of obesity: insulin resistance, improper distribution of body fat, dyslipidemia and the state of meta-inflammation. However, virtually, it is possible that the difference in age between the control and obese group might have had some impact on the values of pro- and antioxidative parameters presented in this study. Such parameters could have been total SOD activity and concentration of MDA, which, according to the literature, are positively correlated with age [48,49,50]. Another such parameter is Cu,Zn-SOD activity and TAC, which were shown to be negatively correlated with age [51,52]. Moreover, Paik et al. [53] showed that the activity of Cu,Zn-SOD could be a marker of zinc supply with diet. Due to this fact, the amount of zinc in the diet might have had some impact on the values of Cu,Zn-SOD. Therefore, the issues covered in this study should be further investigated in future research.

The first factor taken into consideration in this study was the intersex variability of the values of SOD concentration, TAC values, the concentration of MDA and metals: copper, zinc, and cadmium. Men were shown to be characterized by over 1.5 higher concentration of SOD1 and lower SOD3 concentration in plasma, compared to women. The values of TAC and MDA concentration were higher in men. Such an observation seems reliable, since the alterations in TAC are associated with the variability in the concentration of sex hormones [54]. Observations regarding intersexual variability in the concentration of the studied metals are consistent with the results of scientific studies of other authors, in which men have higher zinc concentration, the ratio of zinc to copper, and lower copper, serum, and cadmium in whole blood, in relation to women [55,56,57,58]. The literature also indicates increased accumulation of cadmium by women in relation to men [59,60,61], which coincides with the observations made in this study.

Secondly, two factors, obesity and exposition to cigarette smoke, were taken into consideration. Regardless of sex, markedly increased TAC values were observed in the obese group, although the *p* value in men was slightly above the α (*p* ≈ 0.058). The increase in TAC values could be due to an increase in the concentration of low-molecular-weight antioxidants, such as uric acid, tocopherols, glutathione, and ascorbic acid. The concentration of low-molecular-weight antioxidants was not assayed for in this study. Therefore, the origin of the increase in TAC values observed in this study should be investigated in future research. Interestingly the alterations in values of SOD concentration/activity and the concentration of MDA and metals depended on sex. In women, no difference in SOD concentration/activity was found between the obese and non-obese, although increased MDA, copper concentration, and decreased zinc-to-copper ratio were found in the obese. Conversely, obese men were characterized of increased activity and specific activity of Mn-SOD and no significant differences were observed in values of TAC, MDA, copper concentration, and zinc-to-copper ratio between the obese and non-obese. In both men and women, cadmium concentration was elevated in the obese, although in women this increase was more prominent (therefore statistically significant). Interestingly, in men, SOD1 concentration was increased, and SOD2 concentration was decreased in the obese, compared to the non-obese, although this observation should be investigated further as the *p*-values were ambiguous (*p* ≈ 0.0575 and *p* ≈ 0.0697 regarding SOD1 (ng/mL) and SOD2 (ng/mL), respectively). These findings may indicate that men and women adapt differently to the increased oxidative stress in state of obesity. Other research found in the literature shows a decrease in serum total SOD activity in the obese [62,63,64], an increase total SOD activity in the extracellular fluid [65] and blood [66,67] or no difference in total SOD activity between the obese and non-obese [68]. Decreased Cu,Zn-SOD activity was found in the erythrocytes of the obese [69]. The discrepancy between these results may stem not only from the difference in the type of studied specimen, but also from differences in age of the obese. Karaouzene et al. [70] showed that younger obese individuals were characterized of higher total SOD activity than the older obese individuals, compared to the control groups.

Interesting observations were made in context of exposition to cigarette smoke. In the non-obese, Mn-SOD activity was increased in the exposed. Moreover, the exposed were characterized of markedly (although not clearly significantly, *p* ≈ 0.0592) decreased Cu,Zn-SOD contribution to the total SOD activity pool. In the obese, no such differences were observed. The decrease in Cu,Zn-SOD contribution to the total SOD activity pool may be due to inactivation of Cu,Zn-SOD by the xenobiotics of the cigarette smoke, such as cadmium—a well-known, specific Cu,Zn-SOD inhibitor [26,71] in this study—found to be of increased concentration in individuals exposed to cigarette smoke, regardless of obesity. Elevation of Mn-SOD activity may be an adaptive mechanism to increased oxidative stress, as proposed earlier. The discrepancy between the results obtained in the obese and non-obese, in this context, may indicate a loss of the ability to adapt to cigarette smoke via the increase of Mn-SOD activity in plasma of the obese. The topic of the association between Mn-SOD activity and exposition to cigarette smoke should be further investigated.

Correlations between the studied parameters were analyzed in an attempt to explain some of the observations made in this study. Concentration of copper, cadmium, TAC, and MDA increased with age. The decrease of Cu,Zn-SOD activity with age may be due to inhibition by increasing concentration of cadmium. Increasing glycemia and dyslipidemia observed in obesity were associated with intensification of the process of lipid peroxidation, as shown by the increase of MDA concentration in plasma. As TAC correlated with the same parameters (Table 6) as MDA, and additionally, positively with HOMA-IR, it could be assumed that TAC plays an important role in adaptation to increased oxidative stress due to hyperglycemia, dyslipidemia, and insulin resistance, observed in obesity. Therefore, low-molecular-weight antioxidants, presumably, could support the antioxidative system in the state of increasing inflammation and improper distribution of body fat, in which the total SOD activity is decreased (negative correlation of CRP with total SOD activity, negative correlation of BMI with total SOD activity and Cu,Zn-SOD activity). The lack of correlation between SOD activity and TAC values supports the thesis that enzymatic antioxidants do not significantly contribute to TAC values [72,73,74]. Interestingly, HOMA-IR correlated positively with SOD1 concentration, although no significant correlation was found between HOMA-IR and Cu,Zn-SOD activity. This could be due to lower contribution of SOD1 to Cu,Zn-SOD activity, although the correlation between SOD1 and Cu,Zn-SOD activity, analyzed in this study, was stronger than the correlation between SOD3 and Cu,Zn-SOD activity. The correlation between zinc concentration with TAC values and MDA concentration could be due to covariance between TG and HDL-Chol concentration and zinc concentration, TAC values, and MDA concentration. Therefore, it is likely to be biased. The positive correlation between the concentration of cadmium and Mn-SOD activity accounts for the aforementioned thesis that Mn-SOD plays an important role in the process of adaptation to cigarette smoke. The correlations between SOD1 concentration and SOD3 (positive) and SOD2 (negative) indicate that the three SOD isozymes may be a part of a mechanism which controls their concentration. This interesting issue, covering the origin of the occurrence of intracellular SOD isozymes (SOD1, SOD2) in the plasma and the interaction between the three isozymes, should be further investigated.

All of the aforementioned findings were the basis for analyzing the alterations in concentration/activity of SOD isozymes in context of selected SNPs of genes: *SOD1*, *SOD2*, *SOD3*. One of the selected SNPs (rs8192287, *SOD3*) could not be further analyzed in this study due to the fact that all individuals were of G/G genotype. Rare frequency of genotypes other than G/G is highly probable, since the data from the dbSNP database (National Center for Biotechnology Information) account for rare (≈5.0%) occurrence of the T allele in the European countries.

Interestingly, the C/T genotype of another SNP, the rs4880 (*SOD2*), occurred in more than 90% of the obese group, indicating that this polymorphism might be a hereditary factor for developing obesity. This assumption is supported by the study of Montano et al. [47], in which one of the rs4880 genotypes, Val/Val (T/T), was associated with approximately two-fold increased chance of developing obesity in the Brazilian population. However, compared to our study, a different method, logistic regression, was used, allowing to control the impact of other factors, such as dyslipidemia, and values of systolic blood pressure on the obtained results, and individuals of BMI > 25 were removed from the control group. Logistic regression was not used in this study as only 3 obese individuals out of 44 were of genotype other than C/T. In this case, the maximum likelihood estimation used in logistic regression would be highly biased. In this study, the genotype variability of rs4880 has been found to be associated with alterations in SOD activity, although the statistical analyses could only be performed in the non-obese group, due to the aforementioned dominance of the C/T genotype in the obese group. In the non-obese, the individuals of the T/T genotype were of drastically decreased total SOD activity, Cu,Zn-SOD activity, and Mn-SOD activity in plasma, compared to the individuals of other genotypes (C/C or C/T). These observations support the findings of research by Flekac et al. [40], in which the lowest serum total SOD activity was observed in individuals of T/T genotype. It is known that rs4880 is connected with Val16Ala substitution in the mitochondrial targeting sequence of *SOD2* [45]. However, it has not been clarified how this substitution may affect the activity of both Cu,Zn-SOD and Mn-SOD in extracellular fluids. Surprisingly, in the non-obese group, the individuals of T/T genotype were characterized by significantly higher concentration of glucose and insulin and higher HOMA-IR values, supporting the theory of association between the genotypic variability of rs4880 and increased susceptibility to obesity due to a decrease in the activity of SOD isozymes, leading to increased susceptibility to oxidative damage in tissues, such as β pancreatic cells and altering the response of these cells to the glycemic status.

Other interesting observations were made in the context of genotypic variability of rs2234694 (*SOD1*). Non-obese individuals of the rare (frequency ≈ 4.5%, according to the dsSNP) A/C genotype showed decreased SOD3 concentration, whereas the obese individuals of A/C genotype were characterized of lower SOD1 concentration and higher *SOD2* concentration, compared to the individuals of the A/A genotype. This observation is rather surprising, since rs2234694 is located in the intragenic region of *SOD1*. The alterations in SOD2 and SOD3 concentration may be associated with the aforementioned covariance between SOD isozyme concentration in plasma, shown in this study. No significant alterations in the activity of SOD isozymes were observed in the context of rs2234694. It has previously been reported that the C/C genotype (not detected in this study) is associated with lower total SOD activity, but in the diabetic individuals. Since the concentration of SOD isozymes was not assayed for in that study, it is impossible to discuss these observations any further [40], in context of this study. Interestingly, although the distribution of A/A and A/C genotypes, in this study, did not differ in context of obesity, the obese of A/C genotype showed over 1.5-fold lower concentration of insulin and values of HOMA-IR. Perhaps the association of this SNP (rs2234694) with insulin resistance and susceptibility to type two diabetes should be investigated in future research.

## 4. Materials and Methods

### 4.1. Material

The research was carried out in full blood, serum, plasma, and DNA, isolated from full blood. Full blood was obtained from the biobank of the Polish Center for Technology Development (Wrocław, Poland). Full blood was collected with use of Vacutainer set (Becton Dickinson), according to the standard diagnostic procedure. The samples for obtaining the serum were collected in test tubes containing a coagulation activator (BD Vacutainer, cat. no. 368815) and the samples for obtaining the plasma and isolating DNA were collected in test tubes containing K_2_EDTA (BD Vacutainer, cat. no. 367864). Serum or plasma was obtained by centrifuging the full blood in 2000 *g*, for 15 min. The material was portioned and kept in −80 °C, in Eppendorf test tubes (Eppendorf, cat. no. 0030102.002). The research project has been approved by the Bioethical Committee of Wroclaw Medical University (opinion no. KB 256/2019).

### 4.2. Characteristics of the Population Sample and the Criteria for Exclusion from the Study and Division into Groups

The exclusion criteria were inflammatory and metabolic diseases (other than obesity), cardiovascular diseases tumor diseases and simultaneous treatment with more than two types of drugs, regardless of their mechanism of action. The individuals from whom the specimen was collected were volunteers recruited by the Polish Center for Technology Development (Wroclaw, Poland). The specimen and the information regarding age, height and weight, diseases, treatment, and smoking status were collected on consent of the volunteers. The specimen was assayed for basic laboratory medicine parameters, such as: lipidogram, glucose concentration, insulin concentration, C-reactive protein concentration. HOMA-IR and BMI indices were calculated. The values of these parameters are shown in Table 13. All of the obtained biobanked samples were used in the study.

The main grouping factor was obesity, confirmed on the basis of recruitment information and the values of body mass index (BMI). The cut-off BMI value was 30, according to the WHO recommendations. The population sample consisted of 94 individuals, of which 50 individuals, aged 20–57 years (median value 34) were assigned to the control group and 44 individuals aged 24–75 years (median value 47) were assigned to the obese group. The structure of both these groups are shown in Table 13.

The secondary grouping criterion was exposition to cigarette smoke. It was highly probable that some individuals who were chronically exposed to cigarette smoke would not describe themselves as “smokers” during the recruitment process. Therefore, the classification was based on the cut-off value obtained in a logistic regression model, where the dependent variable was smoking declaration (based on the recruitment survey) and the explanatory variable was the concentration of cotinine, one of the main nicotine biotransformation end products, in serum:

(1) $$P\;\left(Y=1|x_{1},\;x_{2},\;\ldots ,\;x_{k}\right)=\frac{e^{\left(-3.248+0.273x\right)}}{1+e^{\left(-3.248+0.273x\right)}}$$

where “*x*” is the concentration of cotinine (ng/mL). The cut-off value of cotinine concentration, for exposition to cigarette smoke, was 10.6 ng/mL, based on the Youden index (0.87) of the obtained ROC curve. This value lies within the cut-off value range (10–20 ng/mL) found in the literature [75,76,77].

### 4.3. Methods

The measurements were performed with use of SPECORD 40 (Analytik Jena, Jena, Germany) or Multiskan GO (ThermoFisher Scientific, Waltham, MA, USA) spectrophotometers, if not stated otherwise. The necessary incubations were performed in MC-01N (Omega Lab, Delhi, India) or StatFax 2200 (Awareness Technology Inc., Palm City, FL, USA).

#### 4.3.1. Selected Markers of Nicotine Exposure, Carbohydrate, and Lipid Metabolism and Inflammation

Serum cotinine concentration was assayed with use of an ELISA kit (Calbiotech, cat. no. CO096D, El Cajon, CA, USA), utilizing polyclonal anti-human cotinine antibodies. Insulin concentration was determined in serum with use of an ELISA kit (Mercodia, cat. no. 10-1113-01, Uppsala, Sweden), utilizing murine monoclonal anti-human insulin antibodies. Serum glucose concentration was assayed for with use of a glucose oxidase kit (Biomaxima, cat. no. 1-033-0400, Lublin, Poland). A glicerol-3-phosphate oxidase method kit (Biomaxima, cat. no. 1-053-0200, Lublin, Poland) was used in serum triglyceride (TG) concentration assay.

Serum total cholesterol (TChol) concentration was determined with use of GPO/PAP kit (Biomaxima, cat. no. 1-023-0200, Lublin, Poland). Chylomicron, LDL and VLDL cholesterol fractions were precipitated (Biomaxima, cat. no. 1-030-0060, Lublin, Poland) and HDL cholesterol (HDL-Chol) was assayed for with use of the total cholesterol kit mentioned above. Since no triglyceride concentration values over 400 mg/dL were observed, the LDL cholesterol (LDL-Chol) concentration was determined with use of the Friedewald formula [78].

Serum C-reactive protein (CRP) concentration was assayed for with use of a turbidimetric kit, utilizing latex beads bound to anti-human CRP antibodies (Bio-Bas, cat. no. 48030).

#### 4.3.2. Concentration and Activity of Superoxide Dismutase Isozymes

Superoxide dismutase (SOD) isozyme concentrations were assayed for in plasma with use of ELISA kits. *SOD1* concentration kit (ThermoFisher Scientific, cat. no. BMS222, Waltham, MA, USA) utilized monoclonal anti-human SOD1 antibodies, whereas SOD2 (Biomatik, cat. no. 07502, Wilmington, DE, USA) and SOD3 (Biomatik, cat. no. 07504, Wilmington, DE, USA) concentration kits utilized polyclonal anti-human SOD2 (or SOD3) antibodies.

Plasma SOD activity was determined with use of xanthine oxidase/tetrazolium salt method kit (Cayman Chemical, cat. no. 706002, Ann Arbor, MI, USA). Two types of activity were assayed for. Total SOD activity was the activity measured without presence of SOD inhibitors, whereas Mn-SOD (SOD2) activity was measured in presence of potassium cyanide (Merck, cat. no. 1.04967.0100, Kenilworth, NJ, USA), a specific Cu,Zn-SOD inhibitor [79,80]. As a result of optimization of the inhibition reaction, the following conditions were used: 25 min of preincubation of plasma with 3 mM potassium cyanide in 25 °C, in volumetric ratio of 1:1. The absorbance values were read after 90 min after initializing the reaction by addition of xanthine oxidase. Cu,Zn-SOD activity, attributed to SOD1 and SOD3, was calculated by subtracting the values of Mn-SOD activity from the values of total SOD activity.

The values of SOD activity in this work are given in four variants: raw values (U/mL), values corrected by total protein concentration (U/g total protein), specific activity (U/mg SOD isozyme(s)), and contribution to the total SOD activity pool (% total SOD activity). Total protein concentration was determined in plasma with use of micro-biuret method [81], in reference to the standard absorbance curve of BSA (Sigma Chemical, cat. no. A-2153, St. Louis, MI, USA).

#### 4.3.3. Total Antioxidative Capacity and Malondialdehyde Concentration

Total antioxidative capacity (TAC) was determined in serum with use of a kit (Cell Biolabs, cat. no. STA-360, San Diego, CA, USA). Uric acid was used as a reference antioxidant, and TAC values are given as uric acid equivalents (UAE). It should be emphasized that TAC is associated mainly with low-molecular-weight antioxidants (tocopherols, glutathione, uric acid, ascorbic acid, inter alia). Enzymatic antioxidants, such as superoxide dismutase, catalase, and glutathione peroxidase do not have an impact on the values of TAC, as described in the literature [72,73,74].

Malondialdehyde (MDA) concentration was assayed in plasma with use of a kit (Sigma-Aldrich, cat. no. MAK085). To increase the sensitivity of the method, MDA-thiobarbituric acid adducts were extracted in *n*-butanol phase with an addition of 5 M NaCl.

#### 4.3.4. Copper, Zinc, and Cadmium Concentration

Copper and zinc concentration in the serum, and cadmium concentration in full blood were assayed for with use of atomic absorption spectroscopy (ASA) in (certified) Atomic Absorption Spectroscopy Laboratory of the Department and Clinic of Internal and Occupational Diseases, Wroclaw Medical University (Wroclaw, Poland). The assays were performed with use of SOLAAR M6 (Solaar House, Cambridge, UK).

#### 4.3.5. Genotyping

DNA was isolated from K_2_EDTA full blood, with use of Syngen Blood/Cell DNA Mini Kit (Syngen Biotech, cat. no. SY221012, Wroclaw, Poland). The isolated DNA was portioned and stored in −80 °C, in PCR test tubes (Genoplast, cat. no. GBPCR-02-NFT, Rokocin, Poland).

Genotyping of five single nucleotide polymorphisms (SNPs): rs2234694 (*SOD1*), rs5746105 (*SOD2*), rs4880 (*SOD2*), rs927450 (*SOD2*), rs8192287 (*SOD3*) was performed with use of PCR-RFLP method. PCR primers were designed in silico, via Primer-BLAST, based on gene sequences found in GenBank (National Center for Biotechnology Information). The following PCR mixes of *Taq* DNA polymerase, dNTPs and MgCl_2_ were used: Gold Hot Start PCR MIX (Syngen Biotech, cat. no. SY550231, Wroclaw, Poland), Silver Taq PCR MIX (Syngen Biotech, cat. no. SY550331, Wroclaw, Poland), NZYTaq II 2x Colourless Master Mix (NZYTech, cat. no. MB35701). The concentration of each primer (Table 14) in the PCR solution was 0.6 µM. The PCR cycle count was 38 in each product amplification. LifeEco (BIOER) thermocycler was used for both the PCR and the restriction reaction.

**Figure A1:**
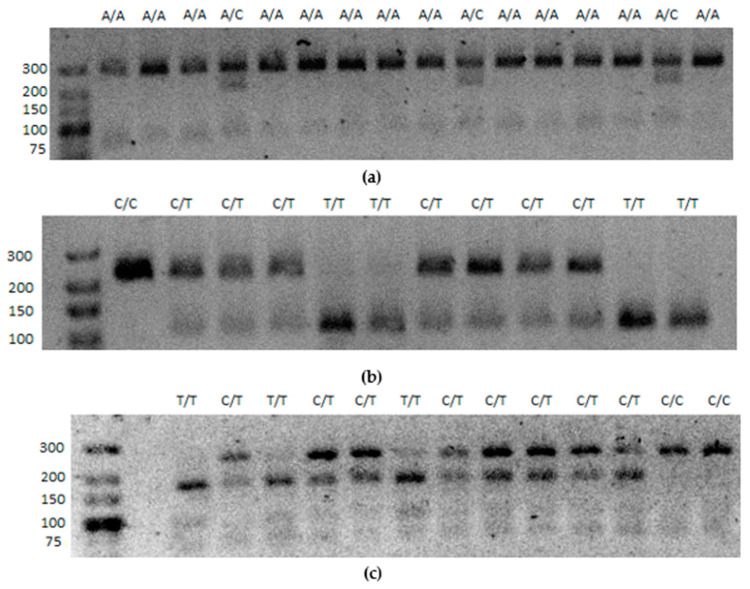
Examples of electropherograms used in the genotyping of: rs2234694 (*SOD1*) (**a**), rs5746105 (*SOD2*) (**b**), rs4880 (*SOD2*) (**c**).

**Figure A2:**
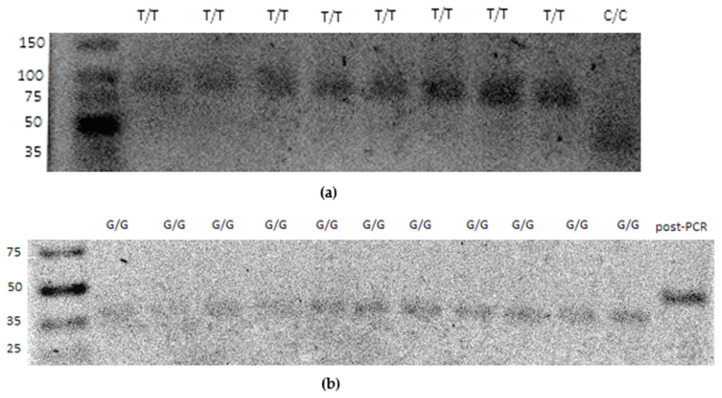
Examples of electropherograms used in the genotyping of: (*SOD2*) (**a**), rs8192287 (*SOD3*) (**b**).

The conditions of restriction reaction are shown in Table 15. Agarose gel electrophoresis of the restriction reaction products was run in 1× TBE buffer, pH 8.2 (Syngen Biotech, cat. no. SY521122, Wroclaw, Poland), in 120V, 60 min, in WHSE-014 and WMHE-600 horizontal gel electrophoresis systems (C.B.S. Scientific), with use of 5.0% Syngen High Resolution Agarose (Syngen Biotech, cat. no. SY521017, Wroclaw, Poland)—for PCR-RFLP of rs8192287 (*SOD3*), or 2.0–4.0% Syngen Daily Agarose (Syngen Biotech, cat. no. SY521011, Wroclaw, Poland)—for PCR-RFLP of rs2234694 (*SOD1*), rs5746105 (*SOD2*), rs4880 (*SOD2*), rs927450 (*SOD2*), rs8192287 (*SOD3*). The following DNA markers were used in electrophoresis: Ultra Low Range DNA Ladder (ThermoFisher Scientific, cat. no. 10597012, Waltham, MA, USA), GeneRuler Ultra Low Range DNA Ladder (ThermoFisher Scientific, cat. no. SM1213, Waltham, MA, USA). Syngen Blue Loading Dye (Syngen Biotech, cat. no. SY521051, Wroclaw, Poland) was used for loading the samples into the wells and Syngen Green DNA Gel Stain (Syngen Biotech, cat. no. SY521031, Wroclaw, Poland) was used for the visualization of DNA in the agarose gel. An exemplary electropherogram for each SNP is shown in Appendix B (Figure A1 and Figure A2).

### 4.4. Statistical Analyses

The obtained data was analyzed with use of STATISTICA 13.3 package, on license provided by Wroclaw Medical University. The nominal α value for hypothesis testing was 0.05. Parametric tests were preferred. Shapiro-Wilk test was used for checking for normality of distribution. Levene test was used to check for equality of variance between the compared groups. In case of normality of distribution and inequality of variances, the t test with Cochran-Cox correction was used for group comparison. In case of lack of normality of distribution, the Mann-Whitney (U) test was used. Monotonic correlations were assessed with use of Spearman’s ρ. Analysis of difference in genotype frequency in context of obesity was performed with use of χ^2^ test, with additional Yates correction of the *p*-value (if any expected count was <5.00).

## 5. Conclusions

In both men and women, obesity is associated with an increase in values of total antioxidative capacity in serum. Additionally, in women, lower zinc-to-copper ratio in serum and higher cadmium concentration in full blood were observed, compared to the non-obese. Increased activity of SOD2 (Mn-SOD) in plasma was observed in obese men.

Exposition to cigarette smoke is associated with an increase in SOD2 (Mn-SOD) activity and decrease in the contribution of Cu,Zn-SOD (SOD1 and SOD3) to the total SOD activity in plasma in non-obese individuals.

Men are characterized of increased SOD1 concentration and decreased SOD3 concentration in plasma, compared to women. These differences coincide with altered values of total antioxidative capacity and concentration of metals: copper, zinc in serum, and cadmium in full blood.

Genotypic variability of SNP rs4880 (*SOD2*) may be associated with obesity, as the C/T genotype was observed in over 90% of obese individuals. The T/T genotype was associated with decreased total SOD activity, Cu,Zn-SOD activity, and Mn-SOD activity in the non-obese.

Genotypic variability of SNP rs2234694 (*SOD1*) is associated with alterations in the concentration of SOD1 and SOD2 in the obese, and in the concentration of SOD3 in the non-obese. These findings may be due to covariance between SOD1 concentration and the concentration both SOD2 (negative) and SOD3 (positive).

## Figures and Tables

**Table 1:** Values of selected pro- and antioxidative parameters, and concentration of selected metals, in context of intersexual variability, in individuals not exposed to cigarette smoke.

Parameter	Women (*n* = 33)	Men (*n* = 35)	*p*
** SOD1 (ng/mL)	{18.14; **23.43**; 33.01}	{35.28; **43.27**; 51.97}	**<0.000001**
** SOD1 (ng/mg total protein)	{0.135; **0.187**; 0.271}	{0.270; **0.348**; 0.442}	**<0.000001**
SOD2 (ng/mL)	{21.80; **23.61**; 32.70}	{20.19; **26.04**; 32.85}	0.9251
SOD2 (ng/mg total protein)	{0.168; **0.203**; 0.262}	{0.152; **0.217**; 0.298}	0.9052
** SOD3 (ng/mL)	{27.71; **31.69**; 37.88}	{23.24; **27.85**; 32.01}	**0.0214**
SOD3 (ng/mg total protein)	{0.195; **0.270**; 0.322}	{0.183; **0.239**; 0.277}	0.0643
SOD (U/L)	{1700; **1971**; 2359}	{1749; **2301**; 2522}	0.2412
SOD (U/g total protein)	{13.20; **16.54**; 20.24}	{12.64; **18.89**; 21.29}	0.3026
SOD (U/mg SODs)	{18.60; **23.30**; 28.20}	{18.03; **22.81**; 25.90}	0.3374
Cu,Zn-SOD (U/L)	{514; **771**; 1106}	{573; **854**; 1184}	0.5977
Cu,Zn-SOD (U/g total protein)	{4.23; **6.07**; 8.64}	{4.35; **6.71**; 10.23}	0.5234
Cu,Zn-SOD (U/mg SOD1+SOD3)	{8.91; **14.29**; 19.03}	{7.76; **12.27**; 15.86}	0.2030
Cu,Zn-SOD (% SOD activity)	{28.60; **40.32**; 55.59}	{32.40; **37.89**; 49.68}	0.5476
Mn-SOD (U/L)	{979; **1226**; 1516}	{1098; **1240**; 1582}	0.5194
Mn-SOD (U/g total protein)	{6.86; **9.99**; 12.33}	{8.60; **10.30**; 12.53}	0.5314
Mn-SOD (U/mg SOD2)	{33.27; **44.54**; 69.62}	{37.76; **50.76**; 66.51}	0.5265
** TAC (mM UAE)	{0.219; **0.268**; 0.308}	{0.345; **0.382**; 0.408}	**<0.000001**
** MDA (µmol/L)	{3.36; **4.43**; 6.60}	{5.29; **6.22**; 7.30}	**0.0085**
** Cu (µg/L)	{976; **1108**; 1303}	{912; **972**; 1059}	**0.0144**
** Zn (µg/L)	{845; **895**; 932}	{961; **1034**; 1116}	**<0.00001**
** Zn/Cu	{0.74; **0.87**; 0.91}	{0.94; **1.03**; 1.18}	**<0.00001**
** Cd (mg/g Hb)	{1.68; **2.46**; 3.56}	{1.12; **1.62**; 2.63}	**0.0125**

Values shown as: 1st quartile; **median value**; 3rd quartile. ** significant difference (median values).

**Table 2:** Values of selected pro- and antioxidative parameters, and concentration of selected metals, in context of obesity, in women not exposed to cigarette smoke.

Parameter	Control Group (*n* = 24)	Obese Group (*n* = 9)	*p*
SOD1 (ng/mL)	**23.45** ± 10.52	**27.54** ± 7.94	0.3002
SOD1 (ng/mg total protein)	**0.184** ± 0.083	**0.236** ± 0.059	0.0982
SOD2 (ng/mL)	{21.16; **23.21**; 31.84}	{22.63; **25.35**; 32.70}	0.4141
SOD2 (ng/mg total protein)	**0.203** ± 0.058	**0.251** ± 0.080	0.0691
SOD3 (ng/mL)	**31.25** ± 8.92	**31.78** ± 4.75	0.8747
SOD3 (ng/mg total protein)	**0.247** ± 0.076^C^	**0.302** ± 0.054	0.0903
SOD (U/L)	**2091** ± 677	**2018** ± 267	0.6667
SOD (U/g total protein)	**16.17** ± 5.91	**16.62** ± 3.29	0.8308
SOD (U/mg SODs)	**25.52** ± 9.57	**21.03** ± 4.74	0.1923
Cu,Zn-SOD (U/L)	**896** ± 516	**720** ± 160	0.1585
Cu,Zn-SOD (U/g total protein)	**6.43** ± 3.80	**6.38** ± 0.91	0.9566
Cu,Zn-SOD (U/mg SOD1+SOD3)	**15.44** ± 7.99	**11.54** ± 4.10	0.0814
Cu,Zn-SOD (% SOD activity)	**43.36** ± 20.30	**36.91** ± 8.22	0.2033
Mn-SOD (U/L)	**1243** ± 558	**1201** ± 261	0.8311
Mn-SOD (U/g total protein)	**9.85** ± 4.58	**10.58** ± 2.88	0.6625
Mn-SOD (U/mg SOD2)	**51.04** ± 28.29	**46.48** ± 19.86	0.6614
* TAC (mM UAE)	**0.252** ± 0.047	**0.353** ± 0.087	**0.0222**
* MDA (µmol/L)	**4.01** ± 1.62	**7.33** ± 1.92	**<0.0001**
* Cu (µg/L)	**1032** ± 174	**1229** ± 91	**0.0087**
Zn (µg/L)	**904** ± 78	**879** ± 130	0.5005
* Zn/Cu	**0.893** ± 0.106	**0.758** ± 0.150	**0.0140**
* Cd (mg/g Hb)	**2.19** ± 0.92	**3.29** ± 1.48	**0.0250**

Values shown as: **mean value** ± standard deviation and {1st quartile; **median value**; 3rd quartile}. * significant difference (mean values).

**Table 3:** Values of selected pro- and antioxidative parameters, and concentration of selected metals, in context of obesity, in men not exposed to cigarette smoke.

Parameter	Control Group (*n* = 17)	Obese Group (*n* = 18)	*p*
SOD1 (ng/mL)	**38.65** ± 11.21	**47.49** ± 13.76	0.0575
SOD1 (ng/mg total protein)	**0.328** ± 0.133	**0.393** ± 0.106	0.1275
SOD2 (ng/mL)	{24.71; **29.83**; 34.94}	{17.84; **22.22**; 29.67}	0.0697
SOD2 (ng/mg total protein)	{0.183; **0.250**; 0.298}	{0.147; **0.187**; 0.273}	0.2968
SOD3 (ng/mL)	**27.09** ± 8.04	**27.46** ± 6.37	0.8857
SOD3 (ng/mg total protein)	**0.222** ± 0.083	**0.240** ± 0.072	0.4982
SOD (U/L)	**2157** ± 611	**2262** ± 330	0.5392
SOD (U/g total protein)	**17.42** ± 6.13	**19.07** ± 3.90	0.3559
SOD (U/mg SODs)	**22.84** ± 7.39	**21.51** ± 5.81	0.5601
Cu,Zn-SOD (U/L)	{530; **1050**; 1301}	{580; **795**; 1071}	0.4827
Cu,Zn-SOD (U/g total protein)	{4.35; **8.98**; 10.77}	{4.79; **6.31**; 9.40}	0.5904
Cu,Zn-SOD (U/mg SOD1+SOD3)	**13.80** ± 5.15	**10.58** ± 4.68	0.0648
Cu,Zn-SOD (% SOD activity)	**41.04** ± 12.11	**36.97** ± 11.61	0.3181
* Mn-SOD (U/L)	**1105** ± 979	**1377** ± 296	**0.0015**
* Mn-SOD (U/g total protein)	**8.90** ± 1.88	**11.97** ± 2.55	**0.0006**
* Mn-SOD (U/mg SOD2)	**44.43** ± 14.80	**59.76** ± 19.18	**0.0145**
TAC (mM UAE)	**0.364** ± 0.043	**0.396** ± 0.053	0.0581
MDA (µmol/L)	**6.04** ± 1.08	**7.01** ± 3.18	0.2350
Cu (µg/L)	**993** ± 168	**983** ± 94	0.8293
Zn (µg/L)	**1038** ± 132	**1022** ± 90	0.6960
Zn/Cu	**1.03** ± 0.17	**1.06** ± 0.17	0.5876
Cd (mg/g Hb)	**1.66** ± 0.97	**2.04** ± 1.14	0.3178

Values shown as: **mean value** ± standard deviation and {1st quartile; **median value**; 3rd quartile}. * significant difference (mean values).

**Table 4:** Values of selected pro- and antioxidative parameters, and concentration of selected metals, in context of exposition to cigarette smoke, in non-obese individuals.

Parameter	Non-Exposed (*n* = 41) (24 Women, 17 Men)	Exposed (*n* = 9) (5 Women, 4 Men)	*p*
SOD1 (ng/mL)	{18.86; **28.56**; 37.42}	{22.52; **30.24**; 42.25}	0.7706
SOD1 (ng/mg total protein)	{0.160; **0.211**; 0.296}	{0.212; **0.242**; 0.379}	0.2788
SOD2 (ng/mL)	{21.73; **25.93**; 33.21}	{21.97; **28.30**; 36.70}	0.7589
SOD2 (ng/mg total protein)	{0.169; **0.210**; 0.268}	{0.168; **0.224**; 0.289}	0.6714
SOD3 (ng/mL)	{25.51; **29.57**; 36.60}	{13.42; **20.66**; 38.77}	0.1879
SOD3 (ng/mg total protein)	{0.179; **0.243**; 0.294}	{0.103; **0.159**; 0.286}	0.1779
SOD (U/L)	{1569; **2290**; 2522}	2087; **2603**; 2803}	0.0910
SOD (U/g total protein)	{12.15; **17.38**; 20.71}	{15.61; **20.52**; 22.62}	0.1926
SOD (U/mg SODs)	{18.80; **24.32**; 30.46}	{17.84; **22.57**; 32.37}	0.9650
Cu,Zn-SOD (U/L)	{495; **854**; 1264}	{607; **847**; 948}	0.6911
Cu,Zn-SOD (U/g total protein)	{4.05; **6.90**; 10.29}	{4.77; **6.26**; 8.42}	0.6193
Cu,Zn-SOD (U/mg SOD1+SOD3)	{9.21; **15.27**; 19.07}	{7.95; **14.90**; 20.60}	0.9185
Cu,Zn-SOD (% SOD activity)	{29.80; **45.53**; 55.59}	{24.57; **30.42**; 33.13}	0.0592
** Mn-SOD (U/L)	{1004; **1199**; 1591}	{1326; **1555**; 2014}	**0.0146**
** Mn-SOD (U/g total protein)	{6.92; **9.97**; 11.66}	{11.05; **11.63**; 15.76}	**0.0419**
Mn-SOD (U/mg SOD2)	{34.94; **42.81**; 65.51}	{33.32; **59.06**; 65.93}	0.6929
TAC (mM UAE)	{0.249; **0.295**; 0.354}	{0.287; **0.304**; 0.347}	0.5321
MDA (µmol/L)	{3.86; **5.02**; 6.18}	{4.40; **5.19**; 5.23}	0.9683
Cu (µg/L)	**1014** ± 170	**1075** ± 90	0.3597
Zn (µg/L)	**951** ± 111	**979** ± 103	0.5348
Zn/Cu	**0.95** ± 0.14	**0.95** ± 0.21	0.9811
** Cd (mg/g Hb)	{1.09; **1.93**; 2.58}	{5.32; **6.30**; 12.12}	**<0.0001**

Values shown as: **mean value** ± standard deviation and {1st quartile; **median value**; 3rd quartile}. ** significant difference (median values).

**Table 5:** Values of selected pro- and antioxidative parameters, and concentration of selected metals, in context of exposition to cigarette smoke, in obese individuals.

Parameter	Non-Exposed (*n* = 27) (9 Women, 18 Men)	Exposed (*n* = 17) (11 Women, 6 Men)	*p*
SOD1 (ng/mL)	{30.44; **37.10**; 49.28}	{27.20; **39.83**; 45.23}	0.5469
SOD1 (ng/mg total protein)	{0.267; **0.328**; 0.426}	{0.206; **0.287**; 0.393}	0.4778
SOD2 (ng/mL)	{19.69; **23.26**; 32.70}	{20.27; **24.52**; 26.62}	0.6636
SOD2 (ng/mg total protein)	{0.151; **0.207**; 0.279}	{0.157; **0.191**; 0.228}	0.4478
SOD3 (ng/mL)	{25.64; **30.02**; 33.84}	{17.34; **27.96**; 31.34}	0.1649
SOD3 (ng/mg total protein)	{0.202; **0.272**; 0.296}	{0.142; **0.243**; 0.277}	0.0853
SOD (U/L)	{1971; **2187**; 2398}	{1723; **2223**; 2477}	1.000
SOD (U/g total protein)	{15.31; **17.55**; 20.78}	{13.75; **18.50**; 20.47}	0.8763
SOD (U/mg SODs)	{17.95; **21.24**; 25.63}	{20.11; **22.95**; 25.73}	0.3488
Cu,Zn-SOD (U/L)	{580; **771**; 1068}	{652; **756**; 852}	0.6540
Cu,Zn-SOD (U/g total protein)	{5.04; **6.06**; 8.90}	{5.06; **5.79**; 6.62}	0.4380
Cu,Zn-SOD (U/mg SOD1+SOD3)	{7.21; **9.62**; 14.29}	{8.92; **10.54**; 13.68}	0.8763
Cu,Zn-SOD (% SOD activity)	{29.94; **37.89**; 42.81}	{28.02; **34.31**; 41.72}	0.3907
Mn-SOD (U/L)	{1105; **1431**; 1495}	{1111; **1460**; 1686}	0.3625
Mn-SOD (U/g total protein)	{9.27; **11.33**; 13.28}	{8.73; **12.07**; 13.85}	0.9243
Mn-SOD (U/mg SOD2)	{37.26; **56.35**; 72.68}	{42.08; **63.77**; 82.98}	0.4668
TAC (mM UAE)	{0.360; **0.386**; 0.426}	{0.330; **0.351**; 0.420}	0.8559
MDA (µmol/L)	{5.83; **6.60**; 9.93}	{6.22; **6.72**; 8.57}	0.3653
Cu (µg/L)	**1061** ± 158	**1103** ± 142	0.3889
Zn (µg/L)	{892; **967**; 1075}	{860; **932**; 1016}	0.3947
Zn/Cu	**0.96** ± 0.22	**0.86** ± 0.16	0.1263
* Cd (mg/g Hb)	**2.45** ± 1.37	**12.54** ± 7.89	**<0.0001**

Values shown as: **mean value** ± standard deviation and {1st quartile; **median value**; 3rd quartile}. * significant difference (mean values).

**Table 6:** Spearman correlation matrix between selected variables, in the entire population sample (*n* = 94).

Variable	Age (years)	TChol (mg/dL)	TG (mg/dL)	HDL-Chol (mg/dL)	LDL-Chol (mg/dL)	CRP (mg/L)	Glucose (mmol/L)	Insulin (mU/L)	BMI	HOMA-IR
Cu (µg/L)	0.25	0.48	0.25		0.37	0.34				
Zn (µg/L)			0.28	−0.42	0.24		0.22			
Cd (mg/g Hb)	0.41								0.23	
SOD1 (ng/mL)			0.32	−0.48				0.28		0.30
SOD2 (ng/mL)										
SOD3 (ng/mL)										
SOD (U/L)		−0.24	−0.26			−0.26		−0.31	−0.21	−0.28
Cu,Zn-SOD (U/L)	−0.37	−0.25			−0.26				−0.26	
Mn-SOD (U/L)										
Cu,Zn-SOD (% SOD activity)	−0.41			0.23	−0.26					
TAC (mM UAE)	0.43		0.30	−0.37		0.32	0.42	0.23	0.59	0.23
MDA (µmol/L)	0.28		0.23	−0.33		0.34	0.27	0.24	0.48	

Significant correlations are colored, depending on direction (red—negative, green—positive). Color saturation depends on the magnitude of correlation (ρ value).

**Table 7:** Spearman correlation matrix between selected variables, in the entire population sample (*n* = 94).

Variable	SOD1 (ng/mL)	SOD2 (ng/mL)	SOD3 (ng/mL)	SOD (U/L)	Cu,Zn-SOD (U/L)	Mn-SOD (U/L)	Cu,Zn-SOD (% SOD activity)	TAC (mM UAE)	MDA (µmol/L)	Cu (µg/L)	Zn (µg/L)	Cd (mg/g Hb)
SOD1 (ng/mL)		−0.29	0.23	0.19	0.33		0.24	0.36				
SOD2 (ng/mL)	−0.29											
SOD3 (ng/mL)	0.23				0.19		0.24					
SOD (U/L)	0.19				0.66	0.64				−0.24		
Cu,Zn-SOD (U/L)	0.33		0.19	0.66			0.78		−0.31			
Mn-SOD (U/L)				0.64			−0.55			−0.30		0.22
Cu,Zn-SOD (% SOD activity)	0.24		0.24		0.78	−0.55			−0.28			
TAC (mM UAE)	0.36								0.33		0.22	
MDA (µmol/L)					−0.31		−0.28	0.33			0.24	

Significant correlations are colored, depending on direction (red—negative, green—positive). Color saturation depends on the magnitude of correlation (ρ value).

**Table 8:** Contingency table of genotype distribution of the selected single nucleotide polymorphisms (SNPs), in context of obesity.

SNP (Gene)	Genotype	Control Group (*n* = 50)	Obese Group (*n* = 44)	*p*
rs2234694 (*SOD1*)	A/A	43 (42.02)	36 (36.98)	0.5807
	A/C	7 (7.98)	8 (7.02)	
rs5746105 (*SOD2*)	C/C	3 (5.32)	7 (4.68)	0.2985
	C/T	24 (22.87)	19 (20.13)	
	T/T	23 (21.81)	18 (19.19)	
**rs4880 (*SOD2*)**	C/C	8 (5.32)	2 (4.68)	**0.0015**
	C/T	31 (38.30)	41 (33.70)	
	T/T	11 (6.38)	1 (5.62)	
rs927450 (*SOD2*)	T/T	16 (15.43)	13 (13.57)	0.8364
	T/C	22 (23.40)	22 (20.60)	
	C/C	12 (11.17)	9 (9.83)	
rs8192287 (*SOD3*)	G/G	50	44	1.0000

Data shown as count (expected count).

**Table 9:** Values of selected pro- and antioxidative parameters, and concentration of selected metals, in context of genotypic variability of rs2234694 (*SOD1*), in non-obese individuals.

Parameter	A/A Genotype (*n* = 43) (26 Women, 17 Men)	A/C Genotype (*n* = 7) (3 Women, 4 Men)	*p*
SOD1 (ng/mL)	{18.86; **29.15**; 42.18}	{24.08; **32.03**; 33.91}	0.7427
SOD1 (ng/mg total protein)	{0.16; **0.23**; 0.34}	{0.18; **0.24**; 0.30}	0.9782
SOD2 (ng/mL)	{22.32; **27.21**; 34.69}	{18.80; **21.97**; 32.99}	0.2090
SOD2 (ng/mg total protein)	{0.17; **0.22**; 0.27}	{0.17; **0.18**; 0.28}	0.3544
** SOD3 (ng/mL)	{25.51; **30.30**; 38.00}	{18.38; **20.66**; 26.04}	**0.0165**
** SOD3 (ng/mg total protein)	{0.18; **0.25**; 0.30}	{0.14; **0.16**; 0.24}	**0.0304**
SOD (U/L)	{1711; **2336**; 2681}	{1528; **2290**; 2553}	0.5649
SOD (U/g total protein)	{13.20; **18.89**; 20.71}	{12.05; **19.47**; 21.40}	0.8268
SOD (U/mg SODs)	{18.33; **23.81**; 30.97}	{22.80; **25.27**; 34.03}	0.3391
Cu,Zn-SOD (U/L)	{532; **847**; 1252}	{411; **1078**; 1264}	0.9346
Cu,Zn-SOD (U/g total protein)	{4.23; **6.77**; 9.94}	{2.86; **8.42**; 11.42}	0.9782
Cu,Zn-SOD (U/mg SOD1+SOD3)	{8.86; **14.79**; 19.07}	{9.51; **18.30**; 26.34}	0.4908
Cu,Zn-SOD (% SOD activity)	{27.92; **35.12**; 54.55}	{27.24; **43.27**; 53.06}	1.0000
Mn-SOD (U/L)	{1004; **1261**; 1676}	{1098; **1145**; 1223}	0.3495
Mn-SOD (U/g total protein)	{8.03; **10.07**; 14.65}	{8.14; **9.51**; 11.05}	0.5465
Mn-SOD (U/mg SOD2)	{33.37; **42.81**; 66.46}	{33.91; **53.65**; 65.93}	0.6714
TAC (mM UAE)	{0.25; **0.30**; 0.35}	{0.27; **0.35**; 0.36}	0.7635
** MDA (µmol/L)	{3.86; **5.02**; 5.83}	{4.48; **6.64**; 7.96}	**0.0423**
Cu (µg/L)	{921; **997**; 1182}	{853; **1059**; 1241}	0.6027
Zn (µg/L)	{846; **929**; 1021}	{902; **965**; 1132}	0.3942
Zn/Cu	{0.81; **0.91**; 1.03}	{0.83; **0.90**; 1.07}	0.9129
Cd (mg/g Hb)	{1.62; **2.43**; 3.51}	{1.12; **1.49**; 5.77}	0.5027
Cotinine (ng/mL)	{0.00; **0.00**; 8.18}	{0.00; **0.00**; 12.54}	0.5649
Age (years)	{24; **34**; 47}	{28; **38**; 49}	0.5649
BMI	{21.36; **23.57**; 26.79}	{20.68; **21.72**; 27.45}	0.7635

Values shown as: **mean value** ± standard deviation and {1st quartile; **median value**; 3rd quartile}. ** significant difference (median values).

**Table 10:** Values of selected pro- and antioxidative parameters, and concentration of selected metals, in context of genotypic variability of rs2234694 (*SOD1*), in obese individuals.

Parameter	A/A Genotype (*n* = 36) (15 Women, 21 Men)	A/C Genotype (*n* = 8) (5 Men, 3 Women)	*p*
** SOD1 (ng/mL)	{31.85; **41.92**; 51.50}	{23.06; **29.06**; 37.58}	**0.0215**
** SOD1 (ng/mg total protein)	{0.27; **0.36**; 0.43}	{0.16; **0.25**; 0.34}	**0.0498**
** SOD2 (ng/mL)	{18.70; **22.63**; 28.03}	{24.01; **27.17**; 35.76}	**0.0460**
** SOD2 (ng/mg total protein)	{0.15; **0.19**; 0.25}	{0.20; **0.23**; 0.30}	**0.0460**
SOD3 (ng/mL)	{23.40; **30.02**; 33.56}	{26.68; **27.75**; 30.84}	0.5095
SOD3 (ng/mg total protein)	{0.18; **0.26**; 0.29}	{0.19; **0.27**; 0.29}	0.9390
SOD (U/L)	{1953; **2198**; 2477}	{1610; **2031**; 2332}	0.4590
SOD (U/g total protein)	{15.31; **18.34**; 20.28}	{12.17; **15.98**; 22.80}	0.6944
SOD (U/mg SODs)	{18.03; **21.98**; 25.73}	{20.82; **21.74**; 24.06}	0.7895
Cu,Zn-SOD (U/L)	{610; **764**; 886}	{390; **713**; 1002}	0.7895
Cu,Zn-SOD (U/g total protein)	{5.18; **6.05**; 7.65}	{3.78; **5.54**; 8.07}	0.6713
Cu,Zn-SOD (U/mg SOD1+SOD3)	{7.86; **10.24**; 13.68}	{8.03; **12.99**; 17.43}	0.3195
Cu,Zn-SOD (% SOD activity)	{29.47; **34.45**; 41.49}	{22.67; **38.93**; 50.30}	0.7654
Mn-SOD (U/L)	{1174; **1441**; 1582}	{886; **1096**; 1551}	0.2477
Mn-SOD (U/g total protein)	{9.48; **11.45**; 13.37}	{7.14; **10.13**; 14.10}	0.5187
Mn-SOD (U/mg SOD2)	{41.91; **59.56**; 75.75}	{24.17; **43.71**; 60.09}	0.1385
TAC (mM UAE)	{0.33; **0.39**; 0.45}	{0.34; **0.38**; 0.44}	0.9390
MDA (µmol/L)	{5.71; **6.82**; 8.69}	{6.16; **6.58**; 9.25}	0.6869
Cu (µg/L)	{945; **1056**; 1154}	{975; **1134**; 1312}	0.2763
Zn (µg/L)	{890; **989**; 1074}	{836; **908**; 944}	0.0622
Zn/Cu	{0.82; **0.94**; 1.07}	{0.72; **0.78**; 0.89}	0.0699
Cd (mg/g Hb)	{1.59; **3.92**; 7.32}	{1.70; **3.43**; 6.15}	0.9860
Cotinine (ng/mL)	{0.00; **0.00**; 15.18}	{0.00; **0.00**; 13.11}	0.8423
Age (years)	{38; **46**; 56}	{39; **52**; 58}	0.7540
BMI	{31.42; **32.29**; 34.25}	{31.57; **32.87**; 34.99}	0.7090

Values shown as: **mean value** ± standard deviation and {1st quartile; **median value**; 3rd quartile}. **—significant difference (median values).

**Table 11:** Values of selected pro- and antioxidative parameters, and concentration of selected metals, in context of genotypic variability of rs4880 (*SOD2*), in **non-obese** individuals.

Parameter	C/C or C/T Genotype (*n* = 39) (24 Women, 15 Men)	T/T Genotype (*n* = 11) (5 Women, 6 Men)	*p*
SOD1 (ng/mL)	{19.78; **32.03**; 42.16}	{16.72; **28.56**; 35.31}	0.5478
SOD1 (ng/mg total protein)	{0.17; **0.24**; 0.35}	{0.13; **0.18**; 0.27}	0.3187
SOD2 (ng/mL)	{22.12; **24.88**; 33.44}	{20.19; **28.11**; 30.25}	0.7105
SOD2 (ng/mg total protein)	{0.17; **0.22**; 0.28}	{0.14; **0.21**; 0.25}	0.5300
SOD3 (ng/mL)	{25.46; **30.02**; 37.88}	{15.70; **22.72**; 29.21}	0.0663
** SOD3 (ng/mg total protein)	{0.18; **0.24**; 0.30}	{0.11; **0.16**; 0.25}	**0.0486**
** SOD (U/L)	{1865; **2359**; 2685}	{1411; **1642**; 1899}	**0.0007**
** SOD (U/g total protein)	{13.39; **20.02**; 21.94}	{9.52; **11.18**; 14.53}	**0.0005**
SOD (U/mg SODs)	{19.32; **23.86**; 32.37}	{17.20; **24.79**; 26.62}	0.4338
** Cu,Zn-SOD (U/L)	{532; **948**; 1301}	{411; **573**; 642}	**0.0202**
** Cu,Zn-SOD (U/g total protein)	{4.23; **8.42**; 10.77}	{2.68; **4.80**; 5.19}	**0.0189**
Cu,Zn-SOD (U/mg SOD1+SOD3)	{10.36; **15.81**; 20.02}	{8.82; **11.81**; 18.17}	0.3618
Cu,Zn-SOD (% SOD activity)	{29.80; **41.26**; 54.55}	{27.24; **31.25**; 49.68}	0.5173
Mn-SOD (U/L)	{1033; **1326**; 1885}	{609; **1155**; 1261}	0.0539
** Mn-SOD (U/g total protein)	{8.81; **10.61**; 15.06}	{5.43; **8.60**; 10.07}	**0.0165**
Mn-SOD (U/mg SOD2)	{35.97; **43.99**; 65.93}	{19.80; **40.36**; 54.32}	0.3618
TAC (mM UAE)	{0.25; **0.30**; 0.35}	{0.22; **0.30**; 0.40}	0.4178
MDA (µmol/L)	{3.86; **5.10**; 5.83}	{4.05; **5.40**; 6.80}	0.3915
Cu (µg/L)	{865; **995**; 1184}	{989; **1021**; 1102}	0.2753
Zn (µg/L)	{853; **929**; 1020}	{895; **992**; 1132}	0.3540
Zn/Cu	{0.81; **0.91**; 1.06}	{0.88; **0.91**; 1.07}	0.9632
Cd (mg/g Hb)	{1.64; **2.43**; 4.26}	{1.03; **2.18**; 2.79}	0.2266
Cotinine (ng/mL)	{0.00; **0.00**; 8.29}	{0.00; **0.00**; 0.00}	0.3302
Age (years)	{25; **33**; 47}	{27; **40**; 49}	0.4178
BMI	{21.15; **22.83**; 26.44}	{23.32; **24.69**; 27.44}	0.0673

Values shown as: {1st quartile; **median value**; 3rd quartile}. **—significant difference (median values).

**Table 12:** The values of selected carbohydrate metabolism and insulin resistance parameters, in context of genotypic variability of rs2234694 (*SOD1*) and rs4880 (*SOD2*).

**rs2234694 (*SOD1*), Obese Group**			
**Parameter**	**A/A Genotype (*n* = 36)**	**A/C Genotype (*n* = 8)**	***p***
Glucose (mmol/L)	{4.94; **5.20**; 5.61}	{4.67; **4.92**; 5.28}	0.2502
** Insulin (mU/L)	{10.00; **14.60**; 19.30}	{7.80; **8.40**; 9.90}	**0.0214**
** HOMA-IR	{2.25; **3.21**; 4.91}	{1.62; **1.75**; 1.85}	**0.0218**
**rs4880 (*SOD2*), Non-Obese Group**			
**Parameter**	**C/C or C/T Genotype (*n* = 39)**	**T/T Genotype (*n* = 11)**	***p***
** Glucose (mmol/L)	{4.50; **4.64**; 4.89}	{4.72; **4.94**; 5.17}	**0.0326**
** Insulin (mU/L)	{4.70; **6.60**; 8.20}	{6.50; **10.00**; 15.20}	**0.0369**
** HOMA	{0.97; **1.35**; 1.84}	{1.28; **2.25**; 3.56}	**0.0306**

Values shown as: {1st quartile; **median value**; 3rd quartile}. ** significant difference (median values).

**Table 13:** The structure and characteristics of groups: control and obese.

**Structure of the Population Sample**				
**Variable (Categorical)**	**Control Group**		**Obese Group**	
Total count	50		44	
Sex	M: 21	F: 29	M: 24	F: 20
Exposed to cigarette smoke	NO: 41	YES: 9	NO: 27	YES: 17
Sex (not exposed)	M: 17	F: 24	M: 18	F: 9
Sex (exposed)	M: 4	F: 5	M: 6	F:11
**Characteristics of the Population Sample**				
**Variable**	**Control Group**		**Obese Group**	***p***
** Age (years)	{25; **34**; 47}		{37; **47**; 57}	**<0.0001**
TChol (mg/dL)	{179; **194**; 215}		{168; **204**; 227}	0.5809
** TG (mg/dL)	{67; **88**; 127}		{87; **134**; 178}	**0.0049**
** HDL-Chol (mg/dL)	{48; **59**; 76}		{43; **53**; 62}	**0.0320**
LDL-Chol (mg/dL)	{99; **117**; 133}		{98; **116**; 142}	0.7743
** CRP (mg/L)	{0.33; **0.65**; 1.10}		{1.11; **1.41**; 3.31}	**<0.00001**
** Glucose (mmol/L)	{4.50; **4.78**; 4.94}		{4.89; **5.17**; 5.61}	**<0.00001**
** Insulin (mU/L)	{4.90; **6.90**; 9.00}		{9.50; **13.95**; 18.00}	**<0.00001**
** BMI	{21.30; **23.48**; 26.79}		{31.51; **32.29**; 34.25}	**<0.00001**
** HOMA-IR	{1.00; **1.49**; 1.94}		{1.85; **3.06**; 4.23}	**<0.00001**

Values shown as: {1st quartile; **median value**; 3rd quartile}. ** significant difference (median values).

**Table 14:** The characteristics of primers used for genotyping (method: PCR-RFLP).

SNP (Gene)	Primers. 5′–3′ Sequence (Base Pair Count)	Melting T (°C)	Annealing T (°C)	GC Content (%)
rs2234694 (*SOD1*)	Forward: CTATCCAGAAAACACGGTGGGCC(23)	64.2	55.0	70.6
	Reverse: TCTATATTCAATAAATGCTACAAAACC(27)	55.9		50.0
rs5746105 (*SOD2*)	Forward: GAGCTCGGTTGATAAAACCAGGG(23)	62.4	58.0	52.2
	Reverse: ACTCAACAAATTTCATAACCCCGA(24)	57.6		37.5
rs4880 (*SOD2*)	Forward: GCCTGCGTAGACGGTCC(17)	60.0	57.0	70.6
	Reverse: TCGGTGACGTTCAGGTTGTT(20)	57.3		50.0
rs927450 (*SOD2*)	Forward: CCTGGAAACCTACATTAAGACTTTG(25)	57.9	57.0	40.0
	Reverse: CTCTGGGGCCTACACTCTTT(20)	58.7		55.0
rs8192287 (*SOD3*)	Forward: TTATGAGTGCGGCTAGTGCC(20)	60.2	57.0	55.0
	Reverse: TACTCGCCCAGTGACAACAC(20)	60.0		55.0

**Table 15:** Restriction conditions (PCR-RFLP method genotyping).

SNP (Gene)	Amplicon Length	Restrictase Restriction Site	Restriction Conditions	Genotype Restriction Fragments
rs2234694 (*SOD1*)	278 bp	HhaI, Thermo Fisher Scientific, cat. no. ER1851	37.0 °C, 10 U HhaI, 1.5 h	A/A: 278 bp
				A/C: 278 bp, 207 bp, 71 bp
				C/C: 207 bp, 71 bp
rs5746105 (*SOD2*)	259 bp	TasI (Tsp509I), Thermo Fisher Scientific, cat. no. ER1351	65.0 °C, 3 U TasI, 2.5 h	C/C: 231 bp, 16 bp, 12 bp
				C/T: 231 bp, 110 bp, 121 bp, 16 bp, 12 bp
				T/T: 110 bp, 121 bp, 16 bp, 12 bp
rs4880 (*SOD2*)	231 bp	BsaWI, New England Biolabs, cat. no. R0567S	60.0 °C, 3 U BsaWI, 2.5 h	C/C: 231 bp
				C/T: 231 bp, 81 bp, 150 bp
				T/T: 81 bp, 150 bp
rs927450 (*SOD2*)	83 bp	BstUI, Thermo Fisher Scientific, cat. no. ER0921	37.0 °C, 3 U BstUI, 2.5 h	T/T: 81 bp
				T/C: 81 bp, 35 bp, 48 bp
				C/C: 35 bp, 48 bp
rs8192287 (*SOD3*)	47 bp	MaeIII, Sigma-Aldrich. cat. no. 10822230001	55.0 °C, 3 U MaeIII, 2.5 h	T/T: 47 bp
				T/G: 47 bp, 32 bp. 15 bp
				G/G: 32 bp, 15 bp

## Abbreviations

BMI	body mass index
GAPDH	glyceraldehyde 3-phosphate dehydrogenase
HOMA-IR	homeostatic model assessment - insulin resistance index
MDA	malondialdehyde
ROS	reactive oxygen species
SNP, SNPs	single nucleotide polymorphism(s)
SOD	superoxide dismutase
SOD1	cytosolic, copper–zinc superoxide dismutase
SOD2	mitochondrial, manganese superoxide dismutase
SOD3	extracellular, copper–zinc superoxide dismutase
SODs	superoxide dismutase isozymes
TAC	total antioxidative capacity
UCP, UCPs	uncoupling protein(s)

## Appendix A

As mentioned before, the first four tables (Table A1, Table A2, Table A3, Table A4) are in context of SNPs: rs5746105 (*SOD2*) and rs927450 (*SOD2*). None of the differences in the first four tables in this appendix were significant, given α = 0.05. The Table A5 is supplementary to the information given in Table 12.

**Table A1:** Values of selected pro- and antioxidative parameters, and concentration of selected metals, in context of genotypic variability of rs5746105 (*SOD2*), in **non-obese** individuals.

Parameter	C/C or C/T Genotype (*n* = 27) (13 Women. 14 Men)	T/T Genotype (*n* = 23) (16 Women. 7 Men)
SOD1 (ng/mL)	{23.43; **33.91**; 42.18}	{18.86; **28.40**; 38.24}
SOD1 (ng/mg total protein)	{0.160; **0.266**; 0.354}	{0.161; **0.212**; 0.324}
SOD2 (ng/mL)	{21.97; **27.55**; 32.85}	{22.34; **24.88**; 36.75}
SOD2 (ng/mg total protein)	{0.168; **0.199**; 0.252}	{0.176; **0.219**; 0.287}
SOD3 (ng/mL)	{20.50; **27.79**; 36.07}	{25.46; **29.21**; 38.12}
SOD3 (ng/mg total protein)	{0.158; **0.232**; 0.286}	{0.184; **0.259**; 0.329}
SOD (U/L)	{1642; **2301**; 2578}	{1602; **2336**; 2763}
SOD (U/g total protein)	{11.61; **17.38**; 21.08}	{13.20; **19.48**; 22.94}
SOD (U/mg SODs)	{20.25; **24.32**; 29.48}	{16.66; **23.31**; 33.93}
Cu,Zn-SOD (U/L)	{495; **948**; 1264}	{530; **780**; 1252}
Cu,Zn-SOD (U/g total protein)	{3.90; **7.04**; 9.94}	{4.05; **6.77**; 10.29}
Cu,Zn-SOD (U/mg SOD1+SOD3)	{8.90; **14.50**; 19.03}	{10.36; **15.86**; 20.02}
Cu,Zn-SOD (% SOD activity)	{27.92; **41.26**; 51.40}	{25.49; **35.12**; 57.02}
Mn-SOD (U/L)	{1118; **1213**; 1591}	{979; **1226**; 1933}
Mn-SOD (U/g total protein)	{8.14; **9.97**; 11.52}	{8.03; **10.08**; 15.92}
Mn-SOD (U/mg SOD2)	{39.10; **49.55**; 64.51}	{27.21; **40.84**; 71.02}
TAC (mM UAE)	{0.266; **0.318**; 0.359}	{0.249; **0.291**; 0.335}
MDA (µmol/L)	{4.05; **5.21**; 6.64}	{3.82; **4.94**; 5.75}
Cu (µg/L)	{972; **1025**; 1232}	{830; **986**; 1155}
Zn (µg/L)	{853; **982**; 1105}	{854; **926**; 1018}
Zn/Cu	{0.81; **0.90**; 1.03}	{0.84; **0.98**; 1.17}

Values shown as: {1st quartile; **median value**; 3rd quartile}.

**Table A2:** Values of selected pro- and antioxidative parameters, and concentration of selected metals, in context of genotypic variability of rs5746105 (*SOD2*), in **obese** individuals.

Parameter	C/C or C/T Genotype (*n* = 26) (13 Women. 13 Men)	T/T Genotype (*n* = 18) (7 Women. 11 Men)
SOD1 (ng/mL)	{29.64; **39.83**; 49.45}	{30.12; **39.47**; 49.28}
SOD1 (ng/mg total protein)	{0.225; **0.334**; 0.437}	{0.274; **0.328**; 0.393}
SOD2 (ng/mL)	{18.17; **22.63**; 28.03}	{21.66; **25.01**; 30.92}
SOD2 (ng/mg total protein)	{0.151; **0.192**; 0.252}	{0.166; **0.211**; 0.279}
SOD3 (ng/mL)	{23.57; **28.96**; 32.98}	{25.60; **30.25**; 32.67}
SOD3 (ng/mg total protein)	{29.64; **39.83**; 49.45}	{30.12; **39.47**; 49.28}
SOD (U/L)	{2016; **2219**; 2419}	{1697; **2045**; 2477}
SOD (U/g total protein)	{16.96; **18.70**; 20.53}	{12.64; **16.55**; 20.47}
SOD (U/mg SODs)	{19.53; **22.39**; 26.49}	{18.03; **20.06**; 25.31}
Cu,Zn-SOD (U/L)	{608; **772**; 1010}	{610; **750**; 852}
Cu,Zn-SOD (U/g total protein)	{5.12; **6.07**; 7.87}	{4.81; **5.98**; 7.65}
Cu,Zn-SOD (U/mg SOD1+SOD3)	{7.48; **11.10**; 13.99}	{8.04; **10.24**; 13.72}
Cu,Zn-SOD (% SOD activity)	{28.65; **34.93**; 46.37}	{29.47; **36.11**; 39.49}
Mn-SOD (U/L)	{1140; **1457**; 1625}	{1047; **1389**; 1460}
Mn-SOD (U/g total protein)	{9.71; **11.78**; 13.87}	{8.96; **10.63**; 12.68}
Mn-SOD (U/mg SOD2)	{42.12; **59.26**; 80.08}	{36.92; **49.58**; 68.37}
TAC (mM UAE)	{0.360; **0.391**; 0.454}	{0.324; **0.377**; 0.426}
MDA (µmol/L)	{6.10; **6.58**; 10.35}	{5.91; **6.82**; 8.46}
Cu (µg/L)	{935; **1056**; 1126}	{960; **1148**; 1308}
Zn (µg/L)	{894; **967**; 1039}	{878; **936**; 1024}
Zn/Cu	{0.79; **0.95**; 1.09}	{0.72; **0.88**; 1.01}

Values shown as: {1st quartile; **median value**; 3rd quartile}.

**Table A3:** Values of selected pro- and antioxidative parameters, and concentration of selected metals, in context of genotypic variability of rs927450 (*SOD2*), in **non-obese** individuals.

Parameter	C/C Genotype (*n* = 12) (8 Women. 4 Men)	T/C Genotype (*n* = 22) (12 Women. 10 Men)	T/T Genotype (*n* = 16) (9 Women. 7 Men)
SOD1 (ng/mL)	{19.32; **33.48**; 37.87}	{20.69; **32.43**; 49.33}	{17.95; **26.22**; 36.78}
SOD1 (ng/mg total protein)	{0.178; **0.241**; 0.326}	{0.161; **0.243**; 0.354}	{0.146; **0.177**; 0.270}
SOD2 (ng/mL)	{22.29; **25.93**; 29.93}	{19.97; **24.31**; 36.72}	{22.34; **28.30**; 33.44}
SOD2 (ng/mg total protein)	{0.186; **0.217**; 0.279}	{0.159; **0.215**; 0.269}	{0.160; **0.203**; 0.289}
SOD3 (ng/mL)	{20.64; **25.73**; 33.03}	{20.84; **29.98**; 40.05}	{18.80; **29.21**; 36.24}
SOD3 (ng/mg total protein)	{0.165; **0.235**; 0.319}	{0.158; **0.250**; 0.310}	{0.160; **0.245**; 0.270}
SOD (U/L)	{2106; **2347**; 2590}	{1602; **2337**; 2685}	{1539; **1882**; 2430}
SOD (U/g total protein)	{15.94; **20.16**; 23.07}	{12.37; **19.18**; 20.71}	{9.97; **13.60**; 20.19}
SOD (U/mg SODs)	{23.81; **27.00**; 32.74}	{17.25; **21.64**; 33.57}	{17.95; **25.27**; 30.50}
Cu,Zn-SOD (U/L)	{604; **1106**; 1388}	{530; **702**; 1105}	{436; **991**; 1164}
Cu,Zn-SOD (U/g total protein)	{4.88; **9.98**; 11.86}	{3.98; **5.73**; 9.68}	{2.72; **7.49**; 8.96}
Cu,Zn-SOD (U/mg SOD1+SOD3)	{11.46; **14.58**; 23.50}	{7.47; **13.52**; 18.25}	{9.51; **17.37**; 19.11}
Cu,Zn-SOD (% SOD activity)	{29.13; **42.88**; 56.25}	{25.49; **33.76**; 50.56}	{29.15; **37.86**; 56.22}
Mn-SOD (U/L)	{1067; **1356**; 1650}	{1004; **1251**; 1885}	{1060; **1182**; 1545}
Mn-SOD (U/g total protein)	{9.40; **11.07**; 12.43}	{8.12; **9.98**; 15.76}	{6.08; **9.60**; 10.94}
Mn-SOD (U/mg SOD2)	{38.05; **48.09**; 65.04}	{30.27; **43.54**; 71.55}	{33.91; **41.48**; 54.32}
TAC (mM UAE)	{0.214; **0.282**; 0.321}	{0.266; **0.296**; 0.345}	{0.265; **0.331**; 0.389}
MDA (µmol/L)	{3.82; **5.06**; 5.98}	{3.86; **5.13**; 6.18}	{3.99; **5.11**; 6.33}
Cu (µg/L)	{904; **976**; 1175}	{853; **1010**; 1184}	{988; **1026**; 1141}
Zn (µg/L)	{821; **854**; 928}	{902; **988**; 1043}	{874; **981**; 1062}
Zn/Cu	{0.75; **0.87**; 1.09}	{0.84; **0.95**; 1.09}	{0.86; **0.91**; 1.03}

Values shown as: {1st quartile; **median value**; 3rd quartile}.

**Table A4:** Values of selected pro- and antioxidative parameters, and concentration of selected metals, in context of genotypic variability of rs927450 (*SOD2*), in **obese** individuals.

Parameter	C/C Genotype (*n* = 9) (4 Women. 5 Men)	T/C Genotype (*n* = 22) (10 Women. 12 Men)	T/T Genotype (*n* = 13) (6 Women. 7 Men)
SOD1 (ng/mL)	{31.89; **43.10**; 50.97}	{27.15; **38.32**; 49.45}	{33.01; **39.83**; 43.27}
SOD1 (ng/mg total protein)	{0.309; **0.356**; 0.426}	{0.215; **0.300**; 0.437}	{0.274; **0.334**; 0.402}
SOD2 (ng/mL)	{21.80; **24.82**; 32.85}	{18.70; **22.70**; 28.03}	{19.69; **23.96**; 28.48}
SOD2 (ng/mg total protein)	{0.185; **0.208**; 0.303}	{0.151; **0.188**; 0.237}	{0.151; **0.194**; 0.262}
SOD3 (ng/m)	{28.16; **31.22**; 33.16}	{20.09; **27.35**; 31.19}	{26.76; **30.60**; 34.42}
SOD3 (ng/mg total protein)	{0.239; **0.274**; 0.291}	{0.147; **0.202**; 0.283}	{0.226; **0.272**; 0.288}
SOD (U/L)	{1697; **1953**; 2351}	{1995; **2128**; 2475}	{1993; **2210**; 2324}
SOD (U/g total protein)	{14.62; **16.99**; 20.47}	{16.55; **18.70**; 21.25}	{14.98; **17.31**; 20.23}
SOD (U/mg SODs)	{16.34; **22.95**; 24.45}	{19.41; **22.09**; 25.99}	{18.60; **21.24**; 25.05}
Cu,Zn-SOD (U/L)	{656; **763**; 804}	{610; **713**; 869}	{580; **774**; 1010}
Cu,Zn-SOD (U/g total protein)	{5.66; **6.54**; 7.05}	{5.05; **5.62**; 7.61}	{4.79; **6.07**; 8.16}
Cu,Zn-SOD (U/mg SOD1+SOD3)	{7.76; **10.10**; 13.99}	{8.07; **10.86**; 14.19}	{7.09; **11.10**; 13.68}
Cu,Zn-SOD (% SOD activity)	{31.95; **34.31**; 38.71}	{28.85; **34.17**; 48.34}	{33.94; **39.49**; 42.81}
Mn-SOD (U/L)	{1134; **1346**; 1460}	{1125; **1441**; 1603}	{1111; **1431**; 1507}
Mn-SOD (U/g total protein)	{9.94; **11.24**; 13.37}	{9.07; **11.70**; 13.87}	{9.27; **11.44**; 12.07}
Mn-SOD (U/mg SOD2)	{41.91; **52.03**; 66.87}	{40.40; **56.72**; 84.90}	{39.70; **61.44**; 72.68}
TAC (mM UAE)	{0.312; **0.391**; 0.405}	{0.331; **0.371**; 0.412}	{0.378; **0.454**; 0.463}
MDA (µmol/L)	{5.98; **6.72**; 8.26}	{4.63; **6.53**; 8.46}	{6.33; **7.65**; 10.74}
Cu (µg/L)	{900; **1183**; 1290}	{1002; **1075**; 1126}	{927; **995**; 1280}
Zn (µg/L)	{881; **952**; 1024}	{900; **999**; 1039}	{788; **932**; 1074}
Zn/Cu	{0.72; **0.89**; 1.02}	{0.79; **0.94**; 1.09}	{0.67; **0.92**; 1.05}

Values shown as: {1st quartile; **median value**; 3rd quartile}.

**Table A5:** Values of selected basic clinical assays: lipidogram, CRP, glucose, insulin concentration and HOMA-IR index, in context of genotypic variability of rs2234694 (*SOD1*) and rs4880 (*SOD2*).

**rs2234694 (*SOD1*) in the Control Group**			
Parameter	**A/A Genotype (*n* = 43) (26 Women, 17 Men)**	**A/C Genotype (*n* = 7) (3 Women, 4 Men)**	***p***
TChol (mg/dL)	{175; **194**; 214}	{184; **194**; 278}	0.2533
TG (mg/dL)	{70; **87**; 127}	{60; **92**; 152}	0.9889
HDL-Chol (mg/dL)	{48; **59**; 76}	{47; **59**; 84}	0.6443
LDL-Chol (mg/dL)	{99; **117**; 130}	{98; **131**; 176}	0.2909
CRP (mg/L)	{0.33; **0.64**; 1.10}	{0.19; **0.93**; 1.20}	0.7220
Glucose (mmol/L)	{4.50; **4.78**; 4.94}	{4.28; **4.55**; 5.28}	0.7058
Insulin (mU/L)	{4.90; **7.25**; 9.00}	{4.70; **5.80**; 10.00}	0.6046
HOMA-IR	{1.02; **1.50**; 1.94}	{0.91; **1.09**; 2.25}	0.6243
**rs2234694 (*SOD1*) in the Obese Group**			
**Parameter**	**A/A Genotype (*n* = 36) (15 Women, 21 Men)**	**A/C genotype (*n* = 8) (5 Men, 3 Women)**	***p***
TChol (mg/dL)	{168; **205**; 228}	{166; **200**; 227}	0.8540
TG (mg/dL)	{84; **118**; 181}	{120; **141**; 162}	0.4158
HDL-Chol (mg/dL)	{43; **53**; 64}	{42; **57**; 60}	0.9413
LDL-Chol (mg/dL)	{98; **115**; 155}	{96; **125**; 142}	0.8829
CRP (mg/L)	{1.03; **1.38**; 3.06}	{1.11; **2.84**; 4.54}	0.6082
Glucose (mmol/L)	{4.94; **5.20**; 5.61}	{4.67; **4.92**; 5.28}	0.2502
**** Insulin (mU/L)**	{10.00; **14.60**; 19.30}	{7.80; **8.40**; 9.90}	**0.0214**
**** HOMA-IR**	{2.25; **3.21**; 4.91}	{1.62; **1.75**; 1.85}	**0.0218**
**rs4880 (*SOD2*) in the Control Group**			
**Parameter**	**C/C or C/T Genotype (*n* = 39) (24 Women, 15 Men)**	**T/T genotype (*n* = 11) (5 Women, 6 Men)**	***p***
TChol (mg/dL)	{179; **197**; 215}	{178; **190**; 242}	0.8041
TG (mg/dL)	{67; **86**; 115}	{75; **102**; 145}	0.3249
HDL-Chol (mg/dL)	{48; **59**; 76}	{46; **62**; 77}	0.6449
LDL-Chol (mg/dL)	{98; **117**; 133}	{99; **118**; 155}	0.9906
CRP (mg/L)	{0.20; **0.64**; 1.05}	{0.41; **0.89**; 1.79}	0.2008
**** Glucose (mmol/L)**	{4.50; **4.64**; 4.89}	{4.72; **4.94**; 5.17}	**0.0326**
**** Insulin (mU/L)**	{4.70; **6.60**; 8.20}	{6.50; **10.00**; 15.20}	**0.0369**
**** HOMA-IR**	{0.97; **1.35**; 1.84}	{1.28; **2.25**; 3.56}	**0.0306**

Values shown as: {1st quartile; **median value**; 3rd quartile}. **—significant difference (median values). Please note, that this table is supplementary to Table 12.

## Appendix B

In this appendix, examples of electropherograms, used in genotyping, are shown (Figure A1 and Figure A2).
